# Potential Effect of the Circadian Clock on Erectile Dysfunction

**DOI:** 10.14336/AD.2021.0728

**Published:** 2022-02-01

**Authors:** Tao Li, Yunjin Bai, Yiting Jiang, Kehua Jiang, Ye Tian, Zhen Wang, Yong Ban, Xiangyi Liang, Guangheng Luo, Fa Sun

**Affiliations:** ^1^Department of Urology, Guizhou Provincial People's Hospital, Guiyang, China.; ^2^Department of Urology and Institute of Urology, West China Hospital, Sichuan University, Chengdu, Sichuan, China.; ^3^Department of Otorhinolaryngology, The Ninth People’s Hospital of Chongqing, Chongqing, China

**Keywords:** circadian clock, disturbed rhythms, penile erection, erectile dysfunction

## Abstract

The circadian rhythm is an internal timing system, which is generated by circadian clock genes. Because the circadian rhythm regulates numerous cellular, behavioral, and physiological processes, organisms have evolved with intrinsic biological rhythms to adapt the daily environmental changes. A variety of pathological events occur at specific times, while disturbed rhythms can lead to metabolic syndrome, vascular dysfunction, inflammatory disorders, and cancer. Therefore, the circadian clock is considered closely related to various diseases. Recently, accumulated data have shown that the penis is regulated by the circadian clock, while erectile function is impaired by an altered sleep-wake cycle. The circadian rhythm appears to be a novel therapeutic target for preventing and managing erectile dysfunction (ED), although research is still progressing. In this review, we briefly summarize the superficial interactions between the circadian clock and erectile function, while focusing on how disturbed rhythms contribute to risk factors of ED. These risk factors include NO/cGMP pathway, atherosclerosis, diabetes mellitus, lipid abnormalities, testosterone deficiency, as well as dysfunction of endothelial and smooth muscle cells. On the basis of recent findings, we discuss the potential role of the circadian clock for future therapeutic strategies on ED, although further relevant research needs to be performed.

With the earth’s rotation, the light and the darkness have a 24-h oscillating cycle [1-4]. To adapt to such environmental light/dark changes, all plants and animals have evolved universally internal circadian rhythms [4], while cues that synchronize intrinsic rhythms with external circumstances are called zeitgebers (time givers) [5]. Numerous cellular, physiological, and behavioral biological processes have shown such rhythmic fluctuations within the 24-h cycle [5]. An example of this rhythmic fluctuation is that blood pressure, heart rate, and body temperature rise in the morning, but decline in the evening [5]. Such inherent rhythms are also observed in sleep [6], diet [7], homeostasis [4, 8], and hormone secretion [4, 8].

During the past 100 years, global industrialization and technological advances have improved modern medical science and promoted human health. However, rising rates of numerous diseases have coincided with altered lifestyles and work patterns [9]. Currently, an increased rate of distant travel, widespread use of artificial light, and modern electronic communication systems have become an essential part in daily life [9]. Elevated work or social pressure, and personal habits with entertainment technological communication platforms have led to widespread use of artificial light or luminescent screens [9]. Insomnia, jet lag, long distance travel across multiple time zones, and prolonged shift work are also more frequent [9]. All of these factors greatly change the daily rest/wake cycles and inevitably chronically disrupt intrinsic circadian rhythms [9]. Numerous studies have shown that disturbed sleep or an impaired circadian clock make individuals more vulnerable to hypertension, type 2 diabetes mellitus (T2DM), hyperlipemia, obesity, atherosclerosis, and cancer (e.g., lung, breast, liver, pancreas, ovary, colon, and prostate cancers) [9-12], meanwhile they also induce oxidative stress, promote inflammatory responses, and accelerate coagulatory responses [13]. All these finally lead to profound problems in people’s health and well-being [9].

Erectile dysfunction (ED) is defined as a consistent or recurrent inability to achieve or maintain a sufficient penile erection for a satisfactory sexual performance [14, 15]. It is a common disease [15, 16] and deteriorates the quality of life (QoL) and sexual satisfaction for men and their partners [17, 18]. According to the National Health and Nutrition Examination Survey, 18 million or 18.4% of men aged >20 years suffer from ED in the USA, and the worldwide prevalence will reach 322 million in 2025 [17]. Although the advance of phosphodiesterase type 5 inhibitor (PDE5i) is a landmark significance for ED, many patients are still refractory or failing to respond as anticipated [19]. Therefore, clarifying the patho-physiological mechanisms of ED is essential before attempting to prevent its progress or improve therapeutic effects.

Accumulating evidence has shown that the penis is also influenced by the circadian clock, while disturbed circadian diseases like sleep disorders, jet leg, and shift working promote ED incidence [11]. Treatment of these diseases can reverse impaired erectile function [19, 20]. Meanwhile, ED is mainly regarded as an organic vascular disease with multifactorial risk factors, such as hypertension, diabetes, hyperlipidemia, and athero-sclerosis [21, 22]; all these factors are closely related to disrupted circadian rhythms [5, 9, 23, 24] (Fig. 1). Recent evidence has also suggested that the progression of ED is regulated by circadian disorders [11, 19, 25, 26].

Herein, we review the relationship between the circadian clock and ED, we also discuss whether and how disturbed circadian rhythms lead to ED from local cellular events (endothelial and smooth cellular function) and systemic factors (diabetes, hyperlipidemia, and atherosclerosis).

**Figure 1.**
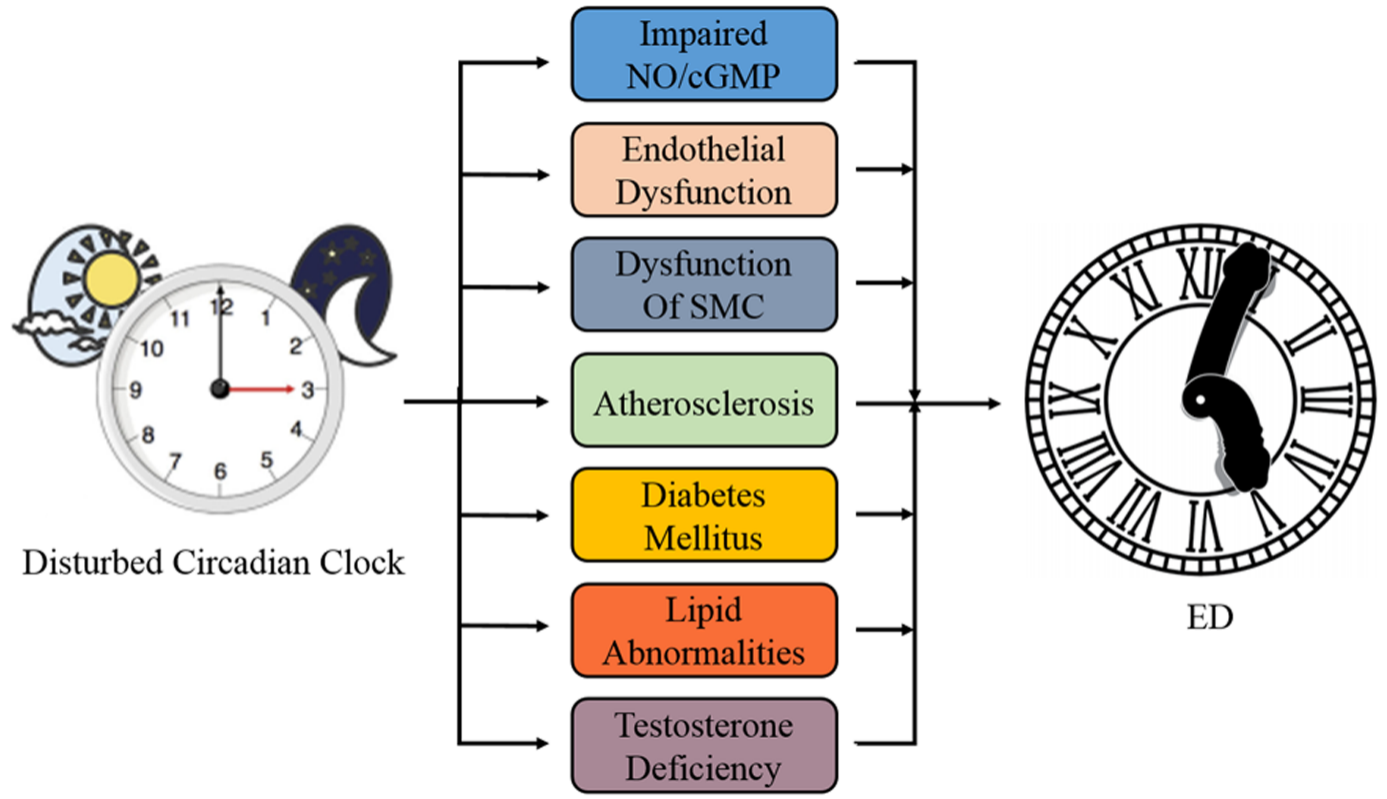
Involvement of circadian clock with ED. The disturbed circadian clock is tightly correlated with NO/cGMP pathway, atherosclerosis, diabetes mellitus, lipid abnormalities, testosterone deficiency, and dysfunction of endothelial and smooth muscle cells, all of which is risk factors of ED. SMC: smooth muscle cell; ED: erectile dysfuncton.

## Biological characteristics of the circadian clock

The central circadian clock is located in the suprachiasmatic nucleus (SCN) of anterior hypothalamus [4]. After photic cues from light-dark cycle are perceived by the retina and transmitted to the SCN as electrical signals, the central circadian system synchronizes with geophysical time [4] and feedbacks to the downstream brain regions and periphery via sympathetic nervous system signaling and hormone release [9, 27]. The synchronization factor, also known as the zeitgeber or time giver, varies from environmental temperature, eating/drinking patterns, pharmacological manipulations, to social interactions; however, light is the fundamental one [4, 28-30]. Additionally, peripheral organs like heart, liver, spleen, and lung also function as “peripheral clocks” and regulate cyclic biological functions by manipulating circadian gene transcription, protein synthesis, and cellular behavior [9]. These “peripheral clocks” are proved as the isolated cells in weeks-long culture still maintained circadian rhythms but can be ceased by serum shock [9, 31]. However, the exact interaction between central and peripheral clocks has not yet been fully clarified [1, 4, 32-34].

**Figure 2.**
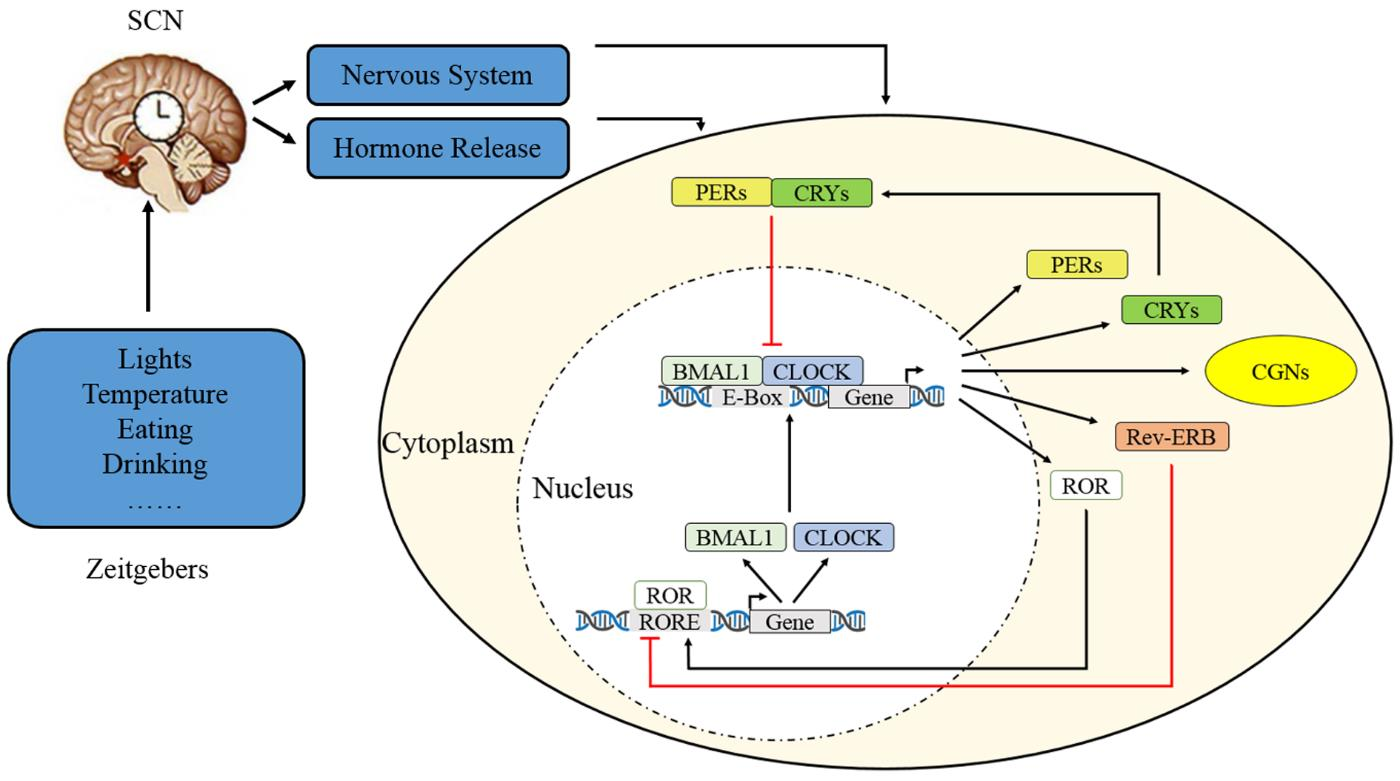
Molecular mechanism of circadian clock. After cues from Zeitgebers of light, temperature, eating, or drinking are perceived and transmitted to SCN as electrical signals, the central circadian clock system synchronizes with geophysical time and feedbacks to the downstream brain regions and periphery via nervous system and hormone release. Briefly, CLOCK and BMAL1 form the core transcription factor of a heterodimer complex and then activates the transcription of other clock genes (like PER, CRY, and REV-ERBα) by integrating with cis-acting element E-box. After entering nucleus, the transcriptional complex of PER and CRY produces a negative feedback loop to repress CLOCK/BMAL1 activity; the PER and CRY are subsequently inhibited while PER/CRY repressor complex also reduced as CLOCK/BMAL1 concentration is decreased. The nuclear receptors of REV-ERBα and RORα are regulated by CLOCK/BMAL1 complex, while REV-ERBα also inhibits BMAL1 transcription but RORα induces it. Thus, the main circadian clock genes are continuously activated by the last with another cycle begins, this auto-regulation feedback loop happens about 24-h.

Approximately 10% of genes are clock-controlled genes (*CCGs*) with circadian oscillations [10, 11, 35]. Mammals are the most complex, with more than 10 clock genes being discovered [10] including circadian locomotor output cycles kaput (*CLOCK*), brain and muscle aryl hydrocarbon receptor nuclear translocator-like (*BMAL1*), the period family (*PER1/2/3*), cryptochrome 1 and 2 (*CRY1/2*), orphan nuclear receptor (*REV-ERBα*), and retinoic acid-related orphan receptor alpha (*RORα*) [4, 9, 10]. These circadian clock genes regulate the day/night fluctuant cycles [4, 10, 36] by positive and negative feedback loops in the SCN and peripheral tissue [9]. Briefly, CLOCK and BMAL1 form the core transcription factor of a heterodimer complex and then activates the transcription of other clock genes (e.g., *PER, CRY,* and *REV-ERBα*) by integrating with cis-acting element E-box [9, 10]. After entering the nucleus, the transcriptional complex of PER and CRY produces a negative feedback loop to repress CLOCK/BMAL1 activity. PER and CRY are subsequently inhibited, while the PER/CRY repressor complex is reduced as CLOCK/BMAL1 concentrations are decreased [9, 10]. The nuclear receptors of REV-ERBα and RORα are regulated by the CLOCK/BMAL1 complex, while REV-ERBα inhibits *BMAL1* transcription and RORα induces it [10, 37] (Fig. 2). Therefore, the main circadian clock genes are continuously activated with another cycle begins, and this auto-regulation feedback loop occurs approximately every 24-h [10, 38].

## Mechanisms of erectile function

Penile erection is a complex neurovascular process, which involves the nervous and endocrine system, as well as endothelial and smooth muscle cells (SMCs) in sinusoids and vessels [39, 40]. The tumescent/erectile or detumescent/flaccid status of the penis is determined by the balance between contractive and relaxant factors [40], and it routinely maintains a flaccid condition with contracted SMCs.

Under sexual stimulation, nitric oxide (NO) is released from nonadrenergic noncholinergic nerve fibers and endothelial cells [14, 18]. NO then diffuses across the smooth muscle membrane to activate guanylate cyclase, which induces protein kinase G (PKG) by increasing cyclic guanosine monophosphate (cGMP) levels. This alteration then decreases cytosolic calcium (Ca^2+^) levels by changing ion channel permeability and finally causes vasodilation of the smooth muscle in the corpora cavernosa [14, 41, 42]. The blood then fills the corpora cavernosa and blocks venous outflow (veno-occlusion) by compressing subcutaneous venules [18]. Therefore, intact endothelial cells and SMCs are fundamental factors for a normal erection. However, the mechanism of ED is complex with diverse risks [14, 18, 43]. With regard to nonendocrine reasons for ED, vasculogenic which affects arterial inflow or venous outflow is the most common (>80% of all cases), others include neurogenic (affecting innervation and nervous function) and iatrogenic (relating to medical or surgical treatment) factors [18]. With regard to endocrine etiologies, reduced serum testosterone levels have been well clarified [18], while the possibility of melatonin is being explored [44].

## Circadian clock and ED

As the main zeitgeber, light is the fundamental synchronization factor that regulates the 24-h circadian cycle [4, 29, 30]. Sleep and wakefulness also tightly co-regulate the circadian clock and the sleep-wake homeostatic process. Therefore, a good sleep rhythm is essential for human health [45]. However, routine behavioral patterns have dramatically altered the day-night rhythm over the past decades [46]. Accumulating evidence has also shown that circadian sleep disorders, disrupted sleep, and insufficient sleep are closely correlated to diverse disease [9-12], including male ED [11, 20, 45] (Fig. 1).

A circadian sleep disorder is defined as an inability to sleep at the desired time rather than sleep generating dysfunction, such as staying up at night for work but sleeping at daytime (shift work disorder) or rapidly traveling to new time zones (jet lag or time zone change syndrome) [45]. For instance, shift work is prevalent worldwide which comprising more than 15% of the workforce [11, 20, 45, 47]. Additionally, up to 50% of some professions, such as police, firefighters, transport drivers, manufacturing employees, and hospital workers, have shift work [45]. An altered sleep-wake cycle inevitably disturbs the internal circadian clock and impairs metabolic homeostasis [5, 8, 9]. These shift workers are likely to feel fatigue, have a lack of energy, and be vulnerable to disease. Meanwhile, they are also unwilling or unable to achieve satisfactory sexual function, especially after shift work at night [5, 45, 48]. Pastuszak et al. studied 182 men and found that nonstandard shift workers had a lower international index of erectile function (IIEF) score, including lower sexual desire, erectile and orgasmic function, as well as intercourse and overall satisfaction [48, 49]. Katherine et al. recruited 745 men and showed the night shift workers showed 7.6 points lower IIEF-EF scores than those worked during the day or evening (P<0.01), while nonstandard shift workers with shift work sleep disorder had lower IIEF-EF (2.8 points) than those without (P<0.01) [11, 20].

As the most important disrupted sleep [26], obstructive sleep apnea (OSA) is defined as partial or complete collapse of the upper airway, and is characterized by loud snoring and absent airflow [45]. It is a common disease with an incidence of 4.0%-32.8% for middle age and 22.4% for older than 60 years [45]. In 1981, Guilleminault et al. initially reported that 48% men with severe OSA suffered from ED [25]. Numerous studies then reported that OSA had a fairly high ED rate (range: 47.1%-80.0%) [45, 50-52] and its severity was correlated with ED development [45, 53]. Additionally, continuous positive airway pressure (CPAP), which is an essential treatment of OSA, significantly improves erectile function [26, 54].

Insufficient sleep is another common sleep disorder that is mainly caused by work schedules and environmental factors like noise and light population [26]. The National Sleep Foundation has recommended a sleep duration of 7-9 h for individuals aged 18-64 years and 7-8 h for those older than 65 years [26, 55]. However, the National Health Interview Survey showed that 30% of workers (approximately 40.6 million) reported an average sleep duration of <6 h in the United States [56]. Insufficient sleep (<5 h) also indicates a lower sexual activity (odds ratio [OR]: 0.88, 95% confidence interval [CI]: 0.80-0.96) and less sexual satisfaction (OR: 0.88, 95% CI: 0.81-0.95), which are improved by extended sleep on the following day [57]. An additional hour of sleep elevates satisfaction in sexual activity by 14% [57].

Although numerous studies have shown that a disturbed sleep rhythm can impair erectile function, the endogenous mechanism has not been clarified. Furthermore, whether and how the circadian clock is involved in erectile dysfunction require further investigation [11, 45].

## Associations between the circadian clock and ED

### Impaired NO/cGMP pathway

As shown previously, NO released from the nerves (nNOS) or endothelium (eNOS) nitric oxide synthase is the primary and indispensable neurotransmitter in penile erection [43], while nNOS initiates penile erection and eNOS maintains or enhances it [40]. The impaired NO production inevitably leads to vasculogenic ED [40]. Actually, old rats with ED always have a lower amount of NOS-containing nerves, as well as decreased NOS mRNA expression and NOS activity [40, 58]. Our previous study also showed that aging rats had lower intracavernous pressure (ICP) and eNOS expression in the corpus cavernosum [59]. Additionally, rats with diabetes or bilateral cavernous nerve crush (BCNC) induced ED showed lower nNOS or eNOS expression and activity, as well as impaired NO production in the corpus cavernosum [39, 40, 60, 61].

The NO/cGMP controlled human forearm blood flow has a peak value at 8 a.m. and a nadir at 8 p.m. [62, 63], this rhythm is also found in the rat aorta and mesenteric arteries [62, 64]. In *Bmal1* knockout mice, superoxide inhibits NO release by suppressing eNOS activation. This inhibition is reversed by scavenging reactive oxygen species (ROS) with PEG-SOD or a non-selective cyclooxygenase inhibitor like indomethacin [9, 65]. The phenylephrine-induced contractile rhythm in the mesenteric artery is abolished in endothelial-specific *Bmal1* knockout [62, 66]. *Per2* mutation mice show decreased NO and vasodilatory prostaglandin production, and enhanced release of vasoconstrictive agents [9, 67]. Insulin-stimulated NO release is also compromised by *Per2* mutation in active and inactive phases [23, 68].

Oxygen is an essential substrate in the NO pathway [25, 69]. Hypoxia impairs eNOS expression and decreases NO concentrations by promoting ROS production (e.g., superoxide, peroxynitrite, and hydrogen peroxide), which finally deteriorates erectile function [19, 25, 69]. For instance, superoxide and peroxynitrite decrease NO concentrations by inducing endothelial cell apoptosis [19, 70, 71], superoxide also reduces NO levels by reacting with it to produce peroxynitrite [19, 71]. Meanwhile, hypoxia promotes vasoconstriction, NO reduction, endothelial dysfunction, and turbulence in the hypothalamic-pituitary-gonadal axis by increasing oxidative stress and sympathetic activity [25]. Because OSA causes intermittent hypoxia, some authors speculate that this circadian disorder impairs erectile function by decreasing NO production, changing sex hormones, and leading to neurological dysfunction [19, 25, 72]. Hypoxia also damages erectile function by inducing vasoconstrictors of endothelin, while CPAP preserves it by attenuating hypoxemia and inhibiting endothelin production in patients with OSA [19, 73]. Lee et al. found that rats in the sleep-deprived group (0.404±0.031) had a significantly lower ICP/MAP ratio than the control (0.718±0.030) and testosterone supplementation (0.55±0.030) groups. Additionally, lower nNOS and eNOS (both mRNA and protein levels) were observed in the sleep-deprived group [11, 74].

Taken together, these studies indicate that the NO/cGMP signal is regulated by circadian variation [62, 63]. However, how the circadian clock affects penile erection and how the NO/cGMP pathway is involved remain to be clarified.

### Endothelial Dysfunction

The penis is a highly vascularized organ, and therefore, penile erection is dependent on the intact endothelial structure of the corpus cavernosum [22]. Briefly, the endothelial bed maintains the erectile process by regulating vascular and smooth muscle vasodilative tone, maintaining vascular pressure, inhibiting thrombosis, and inducing fibrinolysis [75]. Endothelial dysfunction, mainly caused by reduced or absent eNOS-NO bioactivity or bioavailability in the vasculature [75], is defined as decreased responsiveness to vasodilators or increased sensitivity to vasoconstrictors [22, 76]. The endothelial dysfunction is also involved in the circadian clock [23, 62].

*Bmal1* or *Per2* mutation significantly decreases endothelium dependent relaxation of aortic rings [9, 67, 77, 78]. *Bmal1* knockout mice show damaged aortic endothelial function because elevated superoxide concentrations reduce NO production by inhibiting eNOS activation [9, 78]. *Bmal1* knockout or *Clock* mutant mice show endothelial dysfunction and vascular injury by impairing Akt and subsequent NO signaling [9, 77]. In *Per2* mutation mice, endothelium dependent relaxation to acetylcholine in aortic rings is impaired, while transformation of the relaxation response from the inactive to the active phase is decreased in *Per2* mutants, but increased in wild-type littermates [23, 79].

The impaired barrier function, inflammatory response, adhesion molecule expression, and leukocyte migration also contribute to endothelial dysfunction [9, 23, 43, 79, 80]. Genetic *Bmal1* ablation impairs endothelial integrity and barrier function, and promotes expression of chemokines C-C motif ligand (Ccl)8/20 and chemokine (C-X-C motif) ligand (Cxcl)5 [23, 81]. *Clock* promotes intercellular adhesion molecule-1 expression or monocyte adhesion to endothelial cells [23, 82]. By binding to E-box-like enhancer, *Clock* upregulates intercellular adhesion molecule 1 (ICAM-1) expression to promote mononuclear cells adhere to endothelial cells [9, 82]. Meanwhile, sleep-deprived mice show increased pro-inflammatory cytokine expression and decreased CRY1 in vascular endothelial cells. These effects are reversed by the nuclear factor-κB (NF-κB) and cyclic adenosine monophosphate /protein kinase A (cAMP/PKA) pathways after *Cry1* overexpression [23, 83].

The circadian clock also regulates endothelial function by hypercoagulability [9, 23]. The photochemical injured model has shown a diurnal variation in the time of thrombotic vascular occlusion (TTVO), that the TTVO is two-folds higher in the resting phase (zeitgeber time [ZT] 4-8; ZT 0: lights on, ZT 12: lights off) than in the active period (ZT 12-20). This oscillation is disrupted when *Clock*, *Bmal1*, or *Npas2* is deleted or mutated [9, 84]. *Bmal1* regulates the expression of fibrinogen, von Williebrand factor (vWF), and prothrombotic factors plasminogen activator inhibitor (PAI)-1 in aortic endothelial cells [9, 78]. The integral membrane glycoprotein of thrombomodulin, which is essential in regulating intravascular coagulation, also shows a circadian rhythm, while the *Clock:Bmal2* heterodimer elevates its expression by binding to enhance element [9, 85].

Endothelial function is damaged by a disturbed circadian rhythm. But how circadian disorders promote endothelial dysfunction, except for involvement of the NO/cGMP pathway, also needs to be determined.

### Dysfunction of SMCs

Although with normal NO release, a penile erection is absent when the abundance, composition, or regulation of SMCs is altered [22, 86, 87]. Therefore, contraction and relaxation of SMCs in the corpus cavernosum are other central molecular mechanisms of a penile erection [22, 88]. For corpus cavernosum in diabetic ED rat, the maximum SMC contraction in response to phenylephrine is reduced by 50% [22, 89]. Additionally, *in vitro* relaxation of corporal tissue from the penile dorsal artery and the SMC/collagen ratio are reduced, and apoptosis is increased in diabetic rats with ED [22, 90]. Meanwhile, mRNA levels of smooth muscle alpha-actin and differentiated smooth muscle (e.g., smooth muscle myosin heavy chain, smoothelin, calponin, and myocardin) in the corpus cavernosum are also dramatically inhibited [22, 87].

Numerous studies have reported that SMCs are controlled by the circadian clock [9, 23]. In vascular SMCs (VSMCs) of carotid arteries from healthy humans, mRNA expressions of *BMAL1, PER1/2/3*, and *CRY1/2* shows a circadian rhythm. However, this oscillation is attenuated in plaque-derived VSMCs [23, 91]. SMC-specific *Bmal1* knockout mice show decreased blood pressure and compromised circadian rhythm as the vessel contractility is impaired and the arterial lumen diameter is increased. Furthermore, the time-of-day variations in response to agonist-induced vasoconstriction, ROCK2 activation, and myosin phosphorylation are also abolished in mesenteric arteries [23, 92]. In db/bd mice, 24-h mRNA rhythms of *Per1/2* and *Cry1/2* in the aortic and mesenteric arteries, *Per1* and *Rev-erba* in the kidney, and *Per1* in the SCN are significantly suppressed. The contraction-related proteins like Rho kinase 1/2, calponin-3, tropomyosin-1/2, smooth muscle protein 22-α, and PKC-potentiated phosphatase inhibitory protein of 17 kDa are also inhibited [9, 93]. Additionally, 24-h aortic contractile variations in response to phenylephrine (α1-agonist), angiotensin II, and high K^+^ levels are all significantly altered [9, 93]. For VSMCs of thoracic aorta from mice with high-fat diet, Smarcd1 promotes *Bmal1* transcription by directly stimulating and co-activating nuclear *RORα* [9, 94]. Moreover, expression of tissue inhibitor of metalloproteinase 1/3 (timp1/3), collagen 3a1 (col3a1), calponin 1 (cnn1), and transgelin 1 (sm22alpha) in the immortalized VSMC line Movas-1 also shows a circadian pattern [9, 95].

Regardless of the information provided by these studies, how the circadian clock regulates penile erection by manipulating SMC function is unknown. Furthermore, whether a normal circadian rhythm can reverse impaired endothelial cells or SMCs needs to be investigated.

### Atherosclerosis

ED is mainly regarded as an organic vascular disorder [21, 22]. Atherosclerosis, which is an inflammatory disease with leukocyte accumulation [9] and characterized by fatty deposition in arterial inner wall [23], leads to vasculogenic ED [40] by decreasing penile blood flow [96]. As sharing similar risk factors, numerous studies treated ED as a preceded marker of vascular disease to predict cardiovascular disease (CVD) [40, 97, 98]; it is also strongly related to the severity of coronary lesions [22, 97]. For instance, Montorsi et al. found that the incidence of ED was 49% in 300 men with angiographically confirmed coronary artery disease, while 67% of them suffered from ED before coronary artery disease symptoms [22, 99]. A rabbit model with atherosclerotic vascular disease showed that ED incidence was 93% in those with >50% luminal occlusion in the iliohypogastric arteries, while it was only 33% in those with minimal lesions [100, 101]. One explanation for this finding is the arterial size hypothesis, which states that larger coronary vessels can adapt to more narrowing and plaque deposition without obviously reduced blood flow. However, the small cavernosal diameter (<1-2 mm) was more difficult to sustain sufficient blood flow for penile erection, even with minimal luminal narrowing [22].

The Nurses’ Health Study cohort showed that women with 6 years or more of rotating shift work were 1.51 (95% CI: 1.12-2.03) times more likely to develop CVD [24, 102]. Another study found the non-standard shift workers had a 40% higher risk of developing atherosclerosis or CVD than their daytime colleagues [11, 49]. OSA can initiate and accelerate CVD development [25], while sleep disturbance induces arterial atherosclerotic plaques and promotes endothelial dysfunction by inducing inflammation and inflammatory mediators [96, 103, 104]. Moreover, a short sleep duration, poor sleep, and insomnia all lead to CVD by increasing the atherosclerotic risk [96, 105].

Vascular function has a circadian rhythm in healthy mouse aorta [9], as shown by a peak in *Bmal1* expression during the dark phase and a peak in *Per1/2* expression during rest [9, 106]. Disrupted rhythms promote atherosclerotic progression [23] by mediating cardiovascular complications, such as stenotic atherosclerotic lesions, diabetic vasculopathies, senescence, graft failure, and pathological vascular remodeling [9, 46]. Cholesterol ester transfer protein (CETP) mice with a 12-h light-dark shift cycle for 15 weeks show a higher incidence of atherosclerosis [46]. Dominant-negative *Clock* mutant apolipoprotein E (Apoe)^-/-^ mice reveal increased atherosclerosis by enhancing intestinal cholesterol absorption, promoting modified lipoprotein uptake, and decreasing cholesterol efflux from macrophages [9, 107]. In low density lipoprotein receptor (Ldlr)^-/-^ and Apoe-/- mice, *Clock* knockout induces more atherosclerotic lesions at the aortic arches and aortic root [23, 107], but this progress is reduced by upregulated *Cry1* expression [23, 108] or REV-ERBβ agonist delivery [23, 109].

*Bmal1* ablation mice have significantly thickened arterial walls with increased collagen deposition in the medial layer [46, 110]. Accelerated arterial thrombus formation has been found as shown by enhanced von Willebrand factor (vWF), fibrinogen, and plasminogen activation inhibitor-1 (PAI-1) production [46, 111]. *Bmal1* downregulation promotes oxidative stress and this aggravates periodontitis-related atherosclerosis induced by *Porphyromonas gingivalis* [10, 112]. Moreover, aortic grafts from wild-type mice are normal when inserted into *Bmal1^-/-^* or *Per1/2^-/-^* mice. However, aortic grafts from *Bmal1^-/-^* or *Per1/2^-/-^* mice show robust arteriosclerotic disease when transplanted into wild-type mice [9, 113].

*CRY1* mRNA expression is significantly lower in patients with atherosclerosis, while augmented *CRY1* expression reverses the atherosclerotic process by the Toll-like receptor (TLR)/nuclear factor kappa-B (NF-κB) pathway [9, 108]. In hematopoietic cells in Ldlr null mice, decreased *Rev-erbα* expression promotes atherosclerosis, which is suppressed by a synthetic REV-ERB agonist (SR9009) [9, 109]. In *Ldlr^-/-^* mice, *Rev-erbα* knockdown in bone marrow cells increases atherosclerotic lesions around aortic valves. Additionally, inflammatory M1 macrophages are decreased and M2 macrophage markers are increased when *Rev-erbα* is overexpressed in these mice [9, 114].

Taken together, these studies suggest that the circadian clock independently regulates arteriosclerotic disease [9, 113, 114]. However, further clinical and fundamental research is warranted to better clarify the internal molecular pathways in the process of ED.

### Diabetes mellitus

The Massachusetts Male Aging Study showed that men with diabetes had a higher incidence of ED than the general population (28% vs. 9.6%) [40, 115]. Other studies showed that these patients suffered from a 75% lifetime risk of ED and earlier onset than individuals without diabetes [40, 116]. Diabetes mellitus (DM) is the main risk factor for ED [40, 116, 117] because hyperglycemia has systemic effects to impair vasodilatory signaling, and leads to SMC hyper-contractility and veno-occlusive disorder [40, 118, 119]. Diabetes or chronic elevated glucose concentrations decrease NO production by damaging penile nNOS content and activity [40, 120]. This is achieved by inducing the formation of reactive oxygen species (ROS), reactive nitrogen species (RNS) [75], and advanced glycation end products (AGEs) [40, 121]. Glycosylated human hemoglobin impairs smooth muscle relaxation by generating superoxide anions and activating extracellular NO [40, 122], while insulin resistance alters the vascular response to vasoconstriction rather than vasodilation [97]. Furthermore, DM decreases endothelium-dependent vasorelaxation by activating protein kinase C (PCK), impairing cholesterol biosynthesis [22, 123, 124], inhibiting peroxisome proliferator-activated receptor-γ, and increasing the oxidative stress state [22, 89, 125]. All of these factors finally lead to ED [40].

The secretion and sensitivity of insulin display obvious diurnal rhythms [23, 126]. Glucose tolerance is higher in the morning than the evening/night for healthy humans [23, 127], but this rhythm is absent in patients with T2DM because of impaired circadian oscillation of glycometabolism [23, 128]. A comprehensive study of 788 healthy people showed a U-shaped relationship between sleep duration and insulin resistance in which both a long and short sleep duration induced resistance [26, 129]. Five randomized studies indicated that insulin resistance increased by 15%-25% when sleep was restricted for 1, 4, 5, and 14 days [26]. However, this risk was reduced when shift workers improved their sleep duration or had a suitable circadian lifestyle [23, 130]. Indeed, people who experienced social jet lag [26] or shift work [5, 26, 131] suffered from more metabolic diseases such as T2DM and obesity. A prospective study that enrolled 402 nightshift workers and 336 daytime workers showed that nightshift workers had a five-fold higher risk of developing T2DM or obesity after follow up for 4 years [24, 132]. Another meta-analysis that included 34 studies (>2 million individuals) showed that shift workers were associated with higher rates of T2DM and vascular events than non-shift workers [26, 133]. Additionally, circadian rhythm impairment induces insulin resistance in as little as 1 day [26]. This risk increases from 14% to 26% with 1, 2, 3, and 4 days of circadian rhythm impairment, while it is elevated to 55% when combined with sleep restriction [26]. Mice that are exposed to constant 24-h bright light show weight gain and glucose intolerance compared with those with a normal light-dark cycle, although caloric intake is not increased [134].

Numerous fundamental studies have attempted to clarify the relationship between circadian disorders and DM [23]. Impaired glucose tolerance, inhibited insulin secretion, and reduced pancreatic islet proliferation are found in *Clock* [135] or *Bmal1* [136] mutant mice. *Clock* and *Bmal1* mutant mice also develop diabetes by impairing insulin secretion [137]. *Clock* mutation mice have impaired hepatic glycogen oscillation and altered circadian mRNA and protein expression of glycogen synthase 2 (Gys2) (limiting enzyme of glycogenesis) [138]. Damaged glucose tolerance, increased plasma glucose levels, and decreased insulin secretion are found in pancreas- or β-cell-specific *Bmal1* knockout mice [23]. *Bmal1* ablation mice are prone to developing insulin resistance and an obesity phenotype by an alteration in glucose metabolism and impaired insulin signaling [139]. Moreover, *Per2* repression decreases plasma glucose levels, stimulates insulin secretion, and impairs gluconeogenesis [140]. A lack of *Cry1/2* induces glucose intolerance and increases circulating corticosterone levels, suggesting that the hypothalamic-pituitary-adrenal axis may be stimulated and glucocorticoid transactivation is increased [141]. *Rev-erbα* mutant mice with chow diet have increased adiposity (2.5-fold) and mild hyperglycemia (approximately 10%) without insulin resistance [142]. This finding may be explained by REV-ERBα affecting plasma glucose homeostasis by regulating glucose-6-phosphatase and phosphoenol-pyruvate carboxylase [23].

In a word, these findings suggest that the circadian clock regulates glycometabolism by key enzymes of glycometabolism [23, 143]. However, how DM affects circadian disorder and ED is still unclear.

### Lipid abnormalities

Lipid abnormalities, such as decreased high-density lipoprotein (HDL) levels, and increased low-density lipoprotein (LDL) or total cholesterol levels, are well known risk factors for atherosclerosis and endothelial dysfunction, which also finally contribute to ED [100]. The Massachusetts Male Aging Study [100, 115] also showed that low HDL levels were the best predictor of ED. In this study, the incidence of moderate ED increased from 6.7% to 25% for men aged 40 to 55 years, and the incidence of complete ED increased from 0% to 16% in those aged 56 to 70 years when HDL levels decreased from 90 mg/dL to 30 mg/dL [100, 115], while per mmol/L elevated HDL levels indicated a lower risk of ED (0.38 times the risk of ED; 95% CI: 0.18-0.80) [100, 144]. Dietary cholesterol consumption also significantly promoted ED (OR: 1.27, P=0.06), which was marginally reduced by unsaturated fat intake (OR: 0.92, P=0.05) [100, 145]. Lipid abnormalities may reduce endothelium-dependent SMC relaxation in the corpus cavernosum rather than neurogenic-dependent [40, 100, 146]. In hypercholesterolemic animals, endothelium-dependent relaxation is improved with L-arginine, which suggests that a lack of L-arginine causes NO deficiency, but not impaired NOS activity [40, 146].

The circadian clock also has complicated roles in lipid metabolism. Normal circulating lipids show circadian oscillations within a narrow physiological range [9, 23] independent of food intake [23], with peak plasma HDL levels occur in the early rest period and then decrease in the active phase [23, 147]. In *ad libitum*-fed rats/mice with a 12-h photoperiod, plasma cholesterol and triglyceride levels are high in the night owing to a fluctuation in apolipoprotein B lipoprotein levels [9, 148]. Intestinal lipoprotein production shows diurnal variation as absorption of [(3)H]-triolein and [(3)H]-cholesterol is higher at 2400 h than at 1200 h [9]. Protein, mRNA, and activity of microsomal triglyceride transfer protein (MTP) in the intestine and the liver show diurnal variability, which are completely abolished with extended exposure to light or dark [9, 148]. In the liver of *ad libtum*-fed animals, regulators of cholesterol and triglycerides (e.g., sterol regulatory element-binding protein (SREBP)-1c, acetyl co-A carboxylase [ACC], fatty acid synthase [FAS], acetyl-CoA synthase [ACS], 3-hydroxy-3-methylglutaryl-coenzyme A [HMG CoA], and glycerol-3-phosphate acyltranferase [GPAT]) show circadian cycles [9]. In children, a reduced sleep duration increases the risk of overweight [23, 149], while an unhealthy lifestyle or poor sleep is associated with hyperlipidemia and obesity with age [23, 150]. All these studies suggest that circadian rhythms regulate lipid metabolism [23, 151] and that circadian disorders lead to lipid abnormalities [23].

In humans with obesity, 24-h gene expression of *CLOCK*, *BMAL1*, *PER1*, *CRY2*, and *REV-ERBα* in adipocytes is disturbed. Additionally, *CLOCK* expression is related to LDL levels, *RORα* is correlated with HDL levels, and *REV-ERB*α is associated with waist circumference and body mass index (BMI) [23, 152]. Enterocytes in *Clock* mutant mice show increased cholesterol levels, which are absorbed from the intestinal lumen, as well as secretion of chylomicrons and cholesterol [23, 107]. Homozygous *Clock* mutant mice have a significantly attenuated diurnal feeding rhythm, and they develop metabolic syndromes of hyperlipidemia, hyperglycemia, hypoinsulinemia, and hepatic steatosis [9, 135]. Global- or liver-specific *Bmal1* ablation Apoe^-/-^ mice have higher risks of hyperlipidemia and atherosclerosis, which are reversed by virus-mediated *Bmal1* overexpression [23, 153]. Another study also showed that liver-specific *Bmal1* or *Rev-erbα* knockout increased circulating levels of cholesterol, triglycerides, and free fatty acids by accumulating oxidative damage [9, 154, 155]. However, these metabolic outcomes were improved by restoration of hepatic *Bmal1* activity in high fat-fed mice [9, 154]. *Bmal1* knockout increases levels of cholesterol, triglycerides, and free fatty acids by reducing the fat storage capacity in adipose tissue, which finally promotes ectopic fat formation in the liver and skeletal muscle [5, 156]. *PER2* regulates expression of PPARγ and PPARγ target genes to control adipogenesis and lipid metabolism [5]. *Per2* knockout mice show an altered lipid profile and greatly reduced triacylglycerol levels, while fibroblast-specific *Per2* deletion show enhanced adipocyte differentiation [5, 157, 158]. *Per1/2/3* knockout mice are more likely to have obesity, which suggests that it regulates body weight [5, 159]. Liver CYP7A1, which is transcriptionally regulated by the circadian oscillator of NR1D1, promotes cholesterol converse to bile acids, while sleep disturbance accelerates serum and liver cholesterol accumulation by inducing NR1D1-mediated CYP7A1 inhibition [160]. Finally, *Rev-erbα* deletion increases plasma lipid levels, and decreases hepatic cholesterol and triglyceride levels by inducing Insig2 expression [9, 161], while dual depletion of *Rev-erbα* and *Rev-erbβ* function greatly disrupt circadian clock expression and deregulate lipid metabolism [9, 28].

Owing to complex interactions, whether and how the circadian clock regulates ED through lipid metabolism are unclear. Therefore, further studies on this issue are required.

### Testosterone deficiency

Circulating androgen levels are essential for penile maturation and development [22, 162, 163]. Androgen is also necessary in penile erection by regulating the NO/cGMP pathway, activating arterial flow, relaxing corporal smooth muscle, and manipulating veno-occlusion [22, 40]. Penile constitutive NOS activity is impaired in rats with castration or antiandrogen flutamide [22, 40]. Low testosterone levels alter endothelial morphology, change corporal smooth muscle, decrease elastic fibers in the tunica albuginea, and promote extracellular matrix deposition [22, 164, 165]. In older men [22, 166, 167] or patients receiving androgen suppression [22, 166], ED is common as a reduced amount of smooth muscle and increased collagen deposition. However, supplemental testosterone therapy preserves erectile function by reversing corporal structural changes [22, 168, 169], preventing degeneration, and preventing against oxidative damage [11, 74]. Testosterone also decreases visceral obesity and improves the responsive ability to PDE5i [22, 170].

Normal testosterone release has a circadian rhythm, which starts to rise at sleep onset and reaches a peak during the first rapid eye movement (REM) sleep bout [45, 171]. However, this fluctuation is impaired by circadian disruption, such as sleep disturbance and abnormalities of sleep quality or duration [96, 172, 173]. Decreased testosterone levels have been found in patients with insomnia or insufficient sleep [45]. Serum testosterone levels are greatly reduced when sleep is restricted to 5-h per night for 8 nights [45, 174], while salivary testosterone levels decline with acute sleep loss for 33-h [45, 175]. Axelsson et al. studied 42 shift workers and found that dissatisfied staff had lower morning testosterone levels compared with satisfied staff [176, 177]. However, lower testosterone levels were associated with more severe disturbed sleep/wakefulness, greater lack of sleep, and an increased need for recovery after work [176, 177]. Additionally, an increased need for recovery after work was the best predictor of testosterone levels [176, 177]. A study on a 5-day military endurance training course with sleep for 1-3 h showed that testosterone, free testosterone, dehydroepiandrosterone, 17 alpha-hydroxyprogesterone, and androstenedione levels were decreased by 60%-80% [176, 178]. Rats with sleep deprivation for 24-48 h showed reduced serum testosterone levels as a result of 5-HT-related inhibition of testosterone production and reduced testicular StAR protein expression [179].

In summary, the effect of testosterone on erectile function and the circadian clock on testosterone is clear. However, whether and how circadian disruption impairs erectile function by regulating testosterone is unknown, and further research is required.

## Conclusion

Convincing evidence shows that a disturbed circadian clock due to shift work, irregular sleep-wake cycle, or inappropriate modern lifestyle impairs human health and contributes to various diseases, including ED. Accumulated research has also shown that the circadian rhythm is important in affecting several risk factors of ED, such as the NO/cGMP pathway, atherosclerosis, DM, lipid abnormalities, testosterone deficiency, and dysfunction of endothelial and SMCs. However, the mechanism of how the circadian clock regulates erectile function remains elusive, and which specific clock genes are involved in ED also requires further research. There is much to learn about the circadian clock to recommend a healthier lifestyle and a more regular sleep rhythm or duration in humans. This information may provide novel preventative measures and therapeutic targets to reduce the process of ED, and to finally promote sexual satisfaction for men and their partners.
